# Characteristics of immunological events in Epstein-Barr virus infection in children with infectious mononucleosis

**DOI:** 10.3389/fped.2023.1060053

**Published:** 2023-02-09

**Authors:** Yunyun Zhang, Chengrong Huang, Hao Zhang, Zhi Duan, Qian Liu, Jianfei Li, Qiyin Zong, Yu Wei, Futing Liu, Wanlu Duan, Liwen Chen, Qiang Zhou, Qin Wang

**Affiliations:** ^1^ Department of Clinical Laboratory, The Second Hospital of Anhui Medical University, Hefei, China; ^2^ Department of Clinical Laboratory, Anqing Municipal Hospital, Anqing, China

**Keywords:** immunological events, Epstein-Barr virus infection, infectious mononucleosis, children, lymphocytes

## Abstract

**Backgrounds & aims:**

Epstein-Barr virus (EBV) infection occurs commonly in children and may cause acute infectious mononucleosis (AIM) and various malignant diseases. Host immune responses are key players in the resistance to EBV infection. We here assessed the immunological events and laboratory indicators of EBV infection, as well as determined the clinical usefulness of evaluating the severity and efficacy of antiviral therapy in AIM patients.

**Methods:**

We enrolled 88 children with EBV infection. The immune environment was defined by immunological events such as frequencies of lymphocyte subsets, phenotypes of T cells, and their ability to secrete cytokines, and so on. This environment was analyzed in EBV-infected children with different viral loads and in children in different phases of infectious mononucleosis (IM) from disease onset to convalescence.

**Results:**

Children with AIM had higher frequencies of CD3^+^ T and CD8^+^ T cells, but lower frequencies of CD4^+^ T cells and CD19^+^ B cells. In these children, the expression of CD62L was lower and that of CTLA-4 and PD-1 was higher on T cells. EBV exposure induced granzyme B expression, but reduced IFN-*γ* secretion, by CD8^+^ T cells, whereas NK cells exhibited reduced granzyme B expression and increased IFN-*γ* secretion. The frequency of CD8^+^ T cells was positively correlated with the EBV DNA load, whereas the frequencies of CD4^+^ T cells and B cells were negatively correlated. During the convalescent phase of IM, CD8^+^ T cell frequency and CD62L expression on T cells were restored. Moreover, patient serum levels of IL-4, IL-6, IL-10, and IFN-*γ* were considerably lower throughout the convalescent phase than throughout the acute phase.

**Conclusion:**

Robust expansion of CD8^+^ T cells, accompanied by CD62L downregulation, PD-1 and CTLA-4 upregulation on T cells, enhanced granzyme B production, and impaired IFN-*γ* secretion, is a typical characteristic of immunological events in children with AIM. Noncytolytic and cytolytic effector functions of CD8^+^ T cells are regulated in an oscillatory manner. Furthermore, the AST level, number of CD8^+^ T cells, and CD62L expression on T cells may act as markers related to IM severity and the effectiveness of antiviral treatment.

## Introduction

Epstein-Barr (EBV) virus is a human B lymphocytic virus that belongs to the gamma subfamily of herpesvirus (1). EBV is widespread in the population, with 90% of the adults being positive for EBV antibody (2–4). Natural EBV infection, which occurs only in humans, ensues mainly through saliva droplets or close oral contact and can lead to lifelong infection. Current research on EBV infection-associated diseases focuses on infectious mononucleosis (IM), Burkitt lymphoma, diffuse large B-cell lymphoma, nasopharyngeal carcinoma and EBV-associated hemophagocytic lymphohistiocytosis (5). EBV is the common causative agent of the self-limited illness IM that most frequently affects children and adolescents (6).

The host immune response has a critical role in eradicating EBV infection. The main IM pathogenesis is the entry of EBV into the oral cavity and pharynx, where the virus invades cells with CD21 receptors, including oral epithelial cells and B lymphocytes (6). The original surface antigens of infected B lymphocytes are destroyed and deformed, leading to apoptosis, necrosis, or a reduction in the number of B lymphocytes, which triggers a strong T cell immune response (7). EBV-infected cells are known to be controlled by cytotoxic T lymphocytes (8, 9). Loss of CD8 ^+^ T cells led to an increase in the EBV load and IM-like EBV infection in a humanized mice model (10).

Thus, exploring the immunological events associated with EBV infection in IM children is better for investigating the pathogenesis of EBV infection. In our study, these immunological events were analyzed systematically, including the difference in lymphocyte subsets, and phenotypes and function of T cells between children with IM from disease onset to the convalescent phase and healthy controls. Our study involved a systematical analysis of the immune environment of patients with IM and the difference between the different phases of the disease from its onset to convalescence. The analysis allowed us to better comprehend the pathogenesis mechanism and the indicator of disease convalescence during EBV infection.

## Materials and methods

### General information

This study included 88 pediatric patients (58 males and 30 females) who were diagnosed with acute infectious mononucleosis (AIM) at the Second Hospital of the Anhui Medical University between January 2021 and April 2022. None of these children had immunodeficiency, and none had received any immunosuppressive therapy. A total of 30 HCs (in 14 males and 16 females) were also recruited. The Second Hospital Ethics Committee of Anhui Medical University provided its approval for the study protocol. All study participants provided their written, informed consent for participation. All patients with IM met the diagnostic criteria provided by the Chinese Medical Association (CMA) (11). The criteria include any three of the following symptoms: fever, pharyngitis, tonsillitis, enlarged lymph nodes in the neck (more than 1 cm), enlarged liver, and enlarged spleen; Other than that, laboratory indicators show that the lymphocytes count ≥5.0 × 10^9^/L, the atypical lymphocytes count ≥ 1.0 × 10^9^/l or ≥ 10% atypical lymphocytes on a peripheral smear and EBV antibody tests which meet any of the following conditions: (i) VCA-IgM antibody is initially positive and later turns negative; (ii) More than 4-fold increases in VCA-IgG antibody titers compared to paired serum; (iii) EA antibody titer transiently elevated; or (iv) VCA-IgG antibody is initially positive and EBNA antibody later turn positive.

### Detection of serological markers of EBV

Sera were prepared from the peripheral blood. The serum levels of IgG, IgM, and IgA reactivity to different EBV viral antigens were assayed by using the corresponding Chemiluminescence kits (Snibe Diagnostic, Shenzhen, China), as per the manufacturer's instruction. A quantitative real-time polymerase chain reaction was performed to assess the EBV DNA genome loads using a diagnostic kit for EBV DNA (Sansure, Changsha, China). LgEBV DNA was demonstrated by taking the logarithm of 10 for the EBV DNA value.

### Laboratory measurements

Blood samples anticoagulated with EDTA were used for the enumeration of white blood cells, monocytes, lymphocytes, neutrophils, platelets, and hemoglobin levels. The levels of serum alanine aminotransferase (ALT), aspartate aminotransferase (AST), and lactate dehydrogenase (LDH) were measured by the ADVIA Chemistry XPT Automatic Clinical Chemistry Analyzer (Siemens, Germany).

### Flow cytometry

Whole blood samples (100 *μ*l) were collected from each child and stained with antibodies for the flow cytometry analysis. The antibodies used for the lymphocyte subset analysis included FITC-anti-CD3, PE-Cy7-anti-CD19, PE-anti-CD (16 + 56), PerCP-Cy5.5-anti-CD45, APC-Cy7-anti-CD4, Alexa Fluor 750-anti-CD8 (Beckman). For the flow cytometry analysis, surface and intracellular labeling were also performed. The antibodies used for T-cell phenotypes and function analysis included FITC-anti-CD43, PE-anti-CTLA-4, PE-anti-CD44, PE-Cy7-anti-CD62L, and FITC-anti-PD-1 (Biolegend). All cell suspensions were incubated for 20 min at room temperature. Red blood cells were then lysed with the lysing solution, and the cells were then washed and re-suspended in 200ul of PBS. The cells were collected by using the Beckman DxFLEX Flow Cytometer. Data obtained were analyzed with the Kaluza Analysis Software (Beckman Coulter). Based on the scatter signals and with the use of Fixable Viability Dye eFluor 780 (Invitrogen), cell debris and dead cells were removed from the analyses.

### Analysis of the cytokine secretion ability

Freshly isolated lymphocytes from the peripheral blood were stimulated with PMA/ionomycin (Biolegend) combined with Brefeldin A (Invitrogen) for 4 h. The cells were collected after stimulation and stained with the following antibodies: APC-anti-CD16 ^+^ 56, PerCP-Cy5.5-anti-CD3, and Alexa Fluor 750 anti-CD8 (Toimmy Biotech). Using the Intracellular Fixation and Permeabilization Buffer Set (Invitrogen), the cells were fixed, permeabilized, and stained for intracellular cytokine staining with PE-Cy7-anti-IFN*γ* and FITC-anti granzyme B (Toimmy Biotech). The cells were then analyzed with DxFLEX flow cytometry.

In addition, the levels of cytokines IL-2, IL-4, IL-6, IL-10, IFN-*γ*, and IL-17A in the plasma were determined by cytometric bead array (BD Biosciences), and the plasma IL-12 levels were measured by ELISA as per the manufacturer's directions.

### Statistical analyses

Statistical analyses were performed by using the SPSS statistical software (version 22.0, Chicago, IL, United States). The Shapiro-Wilk method was employed for the normality test. For normally distributed data, parametric analysis was applied, and, for non-normally distributed data, non-parametric tests were performed. Correlations were made using Spearman's rank correlation coefficient. Statistical differences were analyzed using a parametric *t*-test or one-way analysis of variance (ANOVA) for multiple comparisons or nonparametric tests. All *P*-values reported were two-sided, and *P* < 0.05 was considered to indicate statistical significance.

## Results

### Patient characteristics

A total of 58 boys and 30 girls with IM (mean age: 3.84 ± 2.46 years; range: 1–13 years) were included as study participants. Compared with the HCs, the counts of WBCs, lymphocytes, and monocytes were significantly increased in children with IM, especially the lymphocyte count. Up to 62.5% of children with IM had ≥10% atypical lymphocytes on a peripheral smear (Table 1). However, the RBC count and hemoglobin level were significantly decreased in children with IM. The serum levels of ALT, AST, and LDH were significantly higher in children with IM than in the HCs (Table 1).

**Table 1:** Basic characteristics of pediatric patients with infectious mononucleosis.

Characteristic	IM (*n* = 88)	HC (*n* = 30)	*P value*
**Mean age (years)**	3.84 ± 2.46	4.47 ± 2.15	0.0941
**Male/Female (*n*)**	58/30	16/14	0.219
Atypical lymphocytes (≥10%)	55 (62.5%)		
EBV VCA lgG(AU/ml)	68 (77.27%)		
EBV VCA lgM(AU/ml)	76 (86.36%)		
EBV VCA lgA (AU/ml)	28 (31.82%)		
EBV EA lgG(AU/ml)	11 (12.5%)		
EBV EA lgA (AU/ml)	35 (49.77%)		
EBV NA lgG(AU/ml)	8 (9.09%)		
Ferritin (ng/ml)	139.17 ± 129.70		
WBC (x 10^9^/L)	13.76 ± 6.69	6.00 ± 1.22	<0.0001
Neutrophils (x 10^9^/L)	3.24 ± 1.84	2.75 ± 0.66	0.3091
Lymphocytes (x 10^9^/L)	8.86 ± 4.87	2.63 ± 0.89	<0.0001
Monocytes (x 10^9^/L)	1.49 ± 1.09	0.39 ± 0.10	<0.0001
Neutrophils (%)	24.31 ± 11.03	46.17 ± 8.22	<0.0001
Lymphocytes (%)	64.16 ± 11.18	43.18 ± 8.23	<0.0001
Monocytes (%)	10.07 ± 4.32	6.49 ± 1.42	<0.0001
RBC (x 109/L)	4.57 ± 0.38	4.74 ± 0.35	<0.05
Hemoglobin (g/L)	122.25 ± 8.86	132.53 ± 12.18	<0.0001
Platelets (x 10^9^/L)	223.05 ± 70.27	285.10 ± 67.29	<0.0001
ALT/ (U/ml)	103.36 ± 133.44	22.50 ± 11.75	<0.0001
AST/ (U/ml)	76.02 ± 72.17	28.14 ± 9.18	<0.0001
LDH/ (U/ml)	435.25 ± 118.17	209.71 ± 47.29	<0.0001

EBV, Epstein-Barr virus; VCA, viral capsid antigen; EA, early antigen; NA, nuclear antigen; WBC, white blood cells; RBC, red blood cells; ALT, alanine aminotransferase; AST, aspartate aminotransferase; LDH: lactate dehydrogenase.

### Characterization of lymphocyte subsets and T cell phenotypes in peripheral blood

**Figure 1:**
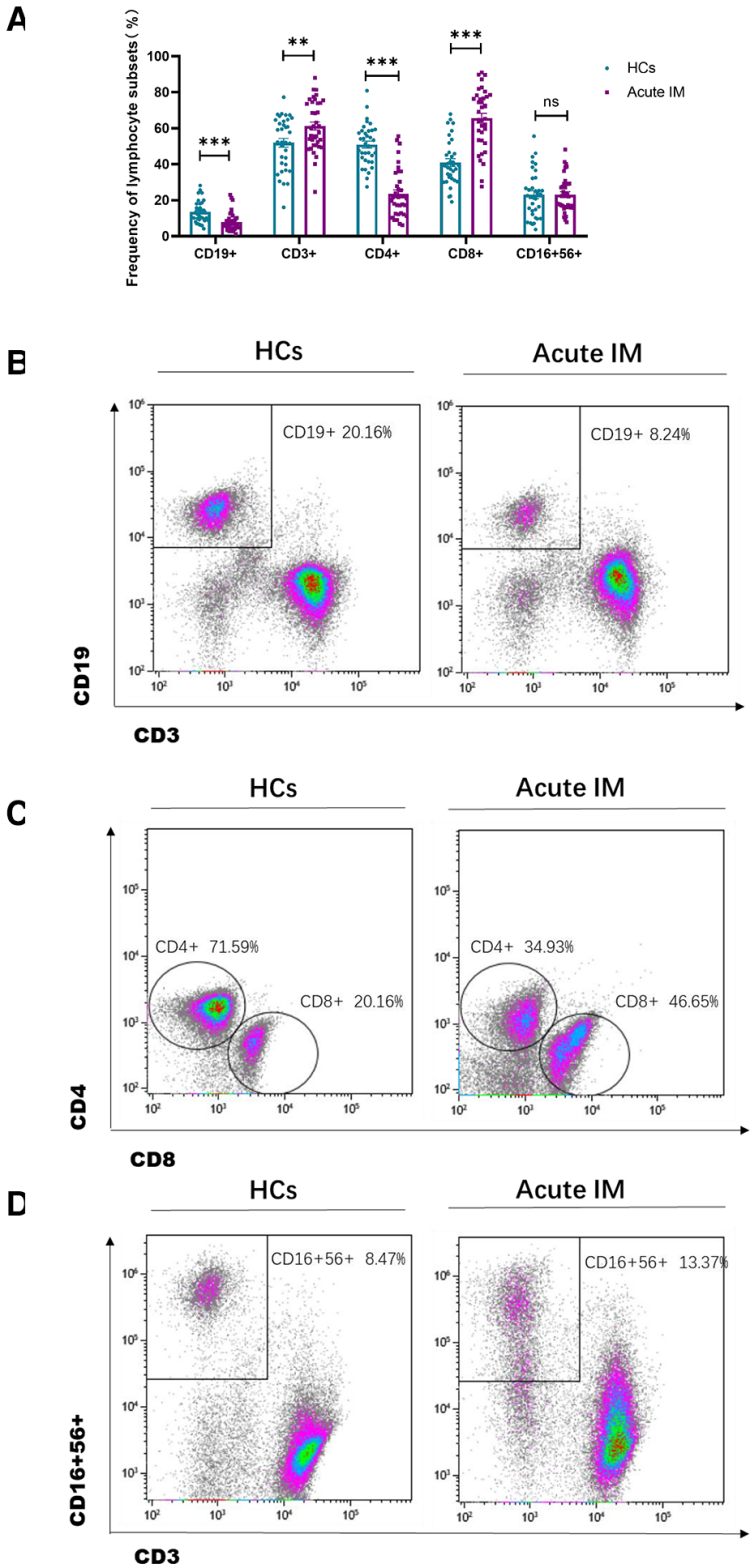
Analysis of the lymphocyte subsets characteristic in pediatric patients with infectious mononucleosis (IM) and in healthy control. (**A**) The frequency of T cells, B cells, and NK cells in patients with IM and healthy control were expressed as the mean with standard deviation (SD). (**B-D**) Representative dot plots showing the frequency of T cells, B cells, and NK cells. ** *P* < 0.01, *** *P* < 0.001.

The lymphocyte subset analysis conducted using peripheral blood revealed that children with IM had considerably higher frequencies and absolute numbers of CD3^+^ and CD8^+^ T cells than HCs (Figures 1A–C, Supplementary Figure S1). However, the frequencies of CD4^+^ T cells and CD19^+^ B cells in patients with IM were substantially decreased (Figures 1A–C), but only a slight variation was observed in absolute cell numbers (Supplementary Figure S1). Meanwhile, the absolute number of NK cells in children with IM increased, yet no discernible difference was noted between the two groups in the frequency of NK cells (Figures 1A,D, Supplementary Figure S1). This suggested that CD8^+^ T cell expansion contributed to lymphocytosis.

**Figure 2:**
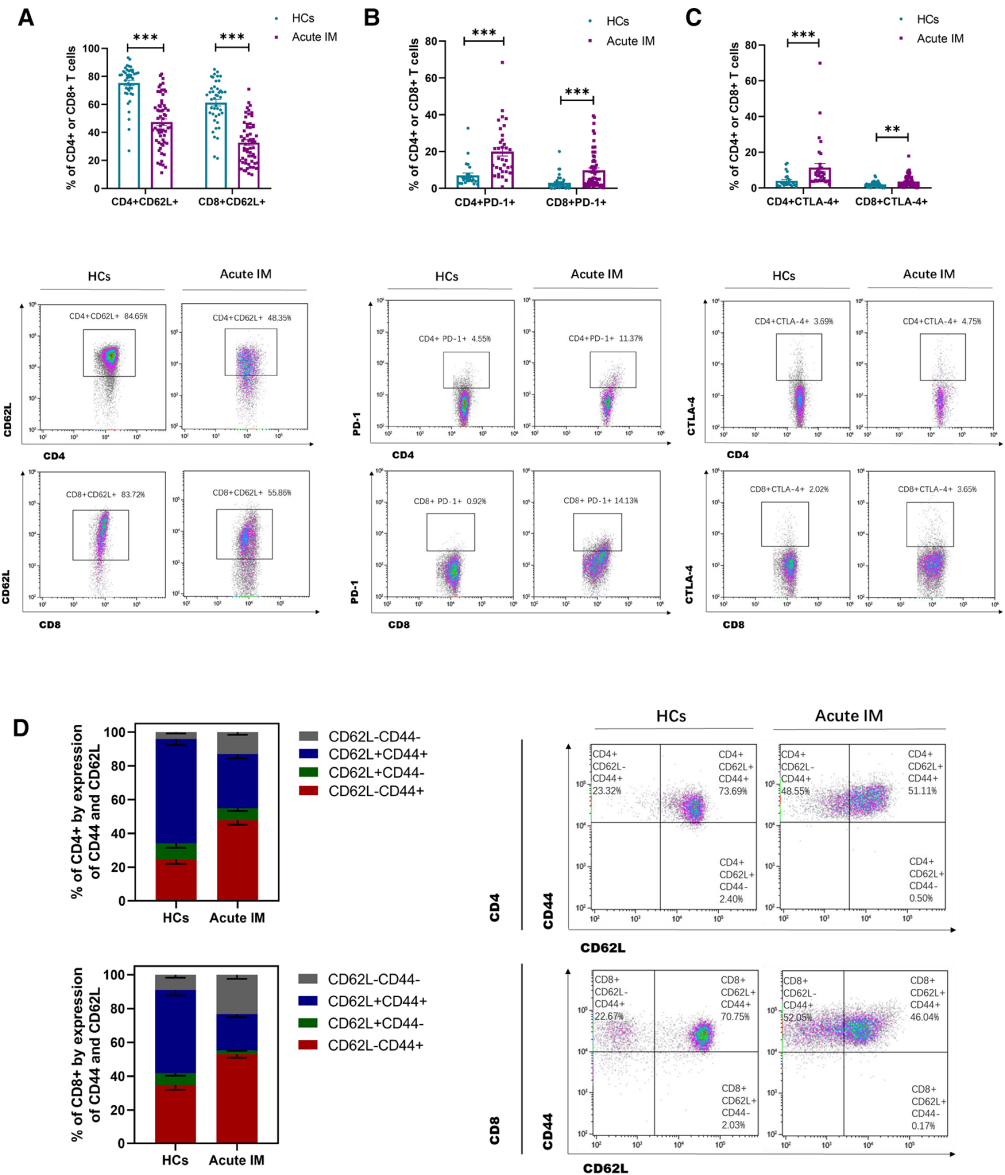
**Analysis of T cell immunophenotype characteristics in pediatric patients with IM.** The activation of T cells was analyzed by flow cytometry, as indicated by active and inhibitory molecules. The expression of (**A**) CD62L, (**B**) PD-1, and (**C**) CTLA-4 on CD4+ and CD8+ T cells in IM patients and HCs. (**D**) The frequencies of the differentiation subsets of CD8+ T cells and CD4+ T cells in the peripheral blood are demonstrated by the CD44 and CD62L expression. Data are expressed as the mean ± SD. ** *P* < 0.01, ***P < 0.001.

By monitoring the expression of activating, stimulatory and inhibitory molecules, immunological phenotypes of T cells, including CD43, CD44, CD62L, PD-1, and CTLA-4, were identified. It was found that CD43 and CD44 were highly expressed on CD4^+^ and CD8^+^ T cells (Supplementary Figure S2). Consistently, children with IM exhibited significant downregulation of CD62L expression on CD4^+^ and CD8^+^T cells in peripheral blood compared with the HCs (Figure 2A). We also examined the expression of the inhibitory molecule PD-1. Interestingly, PD-1 and CTLA-4 expression on CD4^+^ and CD8^+^ T cells was significantly upregulated in children with IM compared with the HCs (Figures 2B,C). Compared with the HCs, the fraction of CD44^+ ^CD62L^−^CD8^+ ^T that accounted for total CD8^+^ T cells and the fraction of CD44 ^+ ^CD62L^−^CD4^+^ T that accounted for total CD4^+^ T cells increased in the peripheral blood of children with IM. This suggested that effector T cells in patients with IM strongly downregulated CD62L, whereas CD44 expression was enhanced (Figure 2D). These results indicated that CD8^+^ and CD4^+^ T cells were strongly activated in children with acute IM; furthermore, they were also regulated by inhibitory molecules to prevent T cell overactivation.

### EBV infection induces CD8^+^ T cell and NK cell immune response

**Figure 3:**
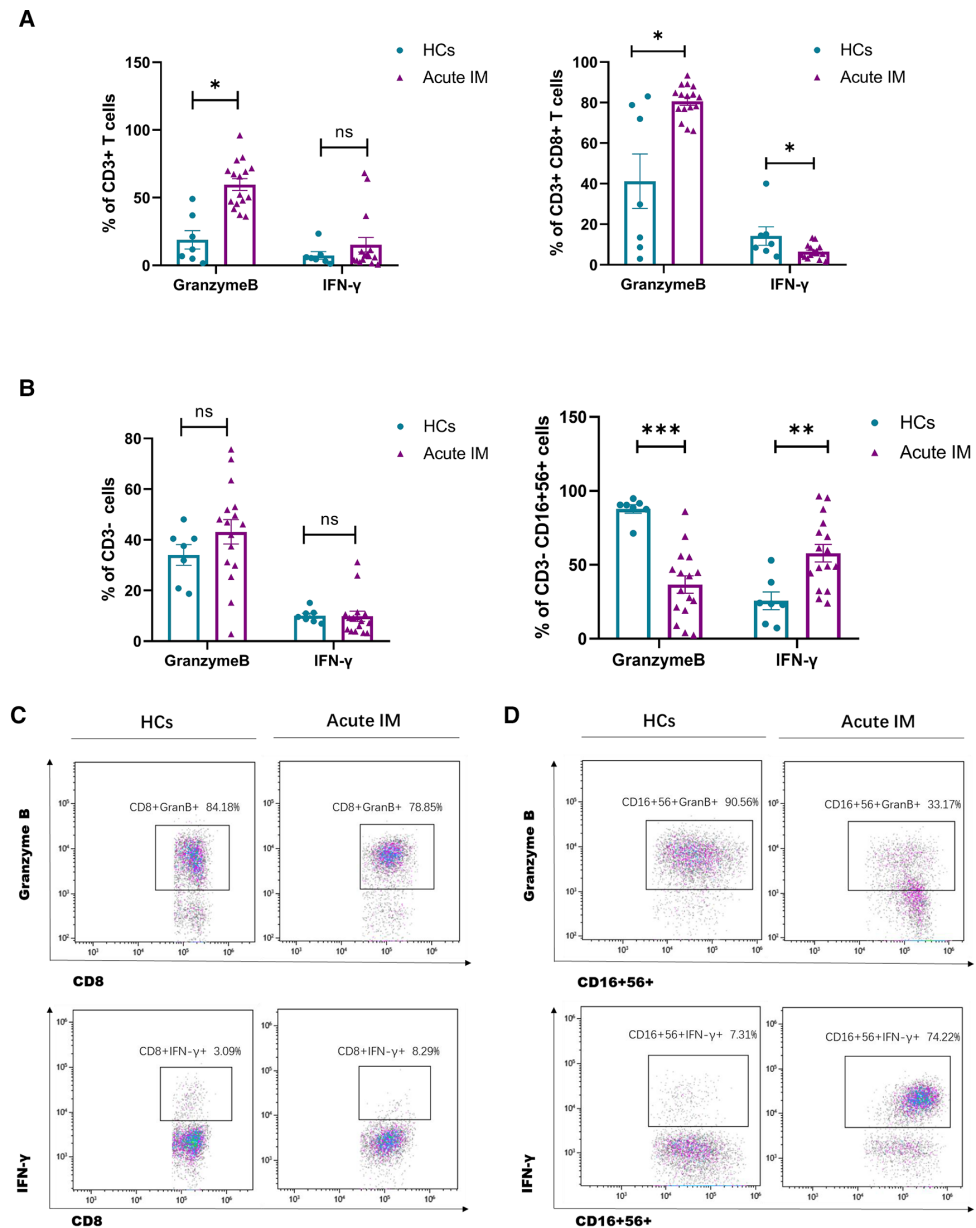
**Analysis of the effector function of CD8^+^ T cells and NK cells.** PBMCs isolated from IM patients and HCs were stimulated by PMA/ionomycin. Intracellular IFN-*γ* and granzyme B staining were performed. The frequency of granzyme B and IFN-*γ* producing CD3^+^ T 、CD3 ^+ ^CD8^+^ T cells (**A**) and CD3^−^、CD3^−^CD16 ^+ ^56^+^ cells **(C**) were measured by flow cytometry. Representative dot plots showing the production of granzyme B and IFN-*γ* in CD8^+^ T cells (**B**) and NK cells (**D**). Data are expressed as the means ± SD. *P < 0.05, **P < 0.01, ***P < 0.001.

To investigate the function of CD8^+^ T cells and NK cells in patients with IM, furthermore, we assessed granzyme B expression and IFN-*γ* secretion by CD8^+^ T and NK cells after stimulation with PMA/ionomycin. The production of granzyme B by CD8^+^ T cells increased significantly, but that by NK cells decreased (Figures 3A–D). By contrast, IFN-*γ* secretion by CD8^+^ T cells decreased, but that by NK cells increased significantly (Figures 3A–D). During viral infection or inflammation, granzyme B is a potent killing factor produced by NK cells and CD8^+^ T cells. In addition, lymphocytes contribute to viral control through noncytolytic effector mechanisms, such as IFN-*γ* production. This result revealed that EBV infection might easily stimulate IFN production and cytolytic activity. A variable antigen burden can induce an oscillation cycle on which antiviral effector activities may be activated and suppressed alternatively (12).

### Different lymphocyte subsets in patients with different EBV loads and their correlation

**Figure 4:**
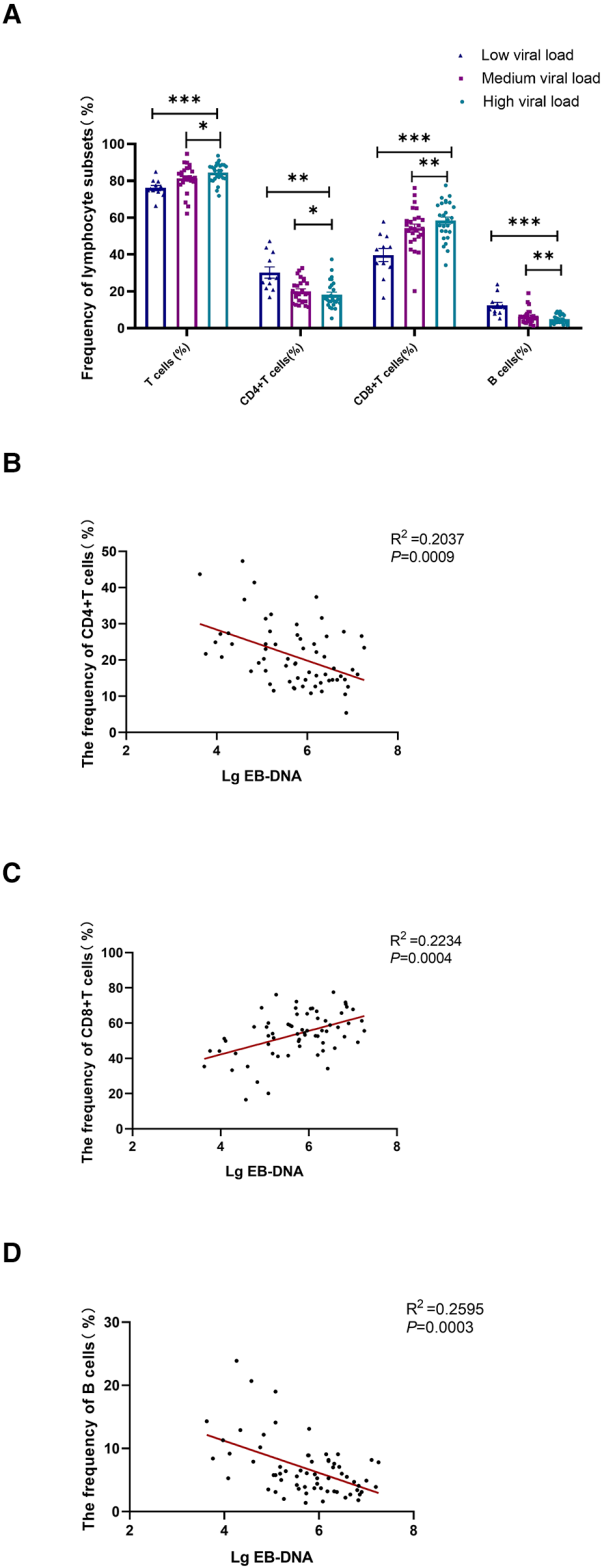
**Correlational analysis between the EB viral load and the frequency of lymphocyte subsets.** Patients with acute IM were categorized into 3 groups based on the EBV viral load. Low viral load represents 2.6 < LgEB-DNA < 5; medium viral load represents 5 ≤ LgEB-DNA < 6; high viral load represents 6 ≤ LgEB-DNA < 8. (**A**) The frequency of T cells, CD4^+^ T cells, CD8^+^ T cells and B cells in different viral load groups. Correlation between EB viral load and the frequency of CD4^+^ T cells (**B**), CD8^+^ T cells (**C**) and B cells (**D**) were displayed. Data are expressed as the means ± SD. *P < 0.05, **P < 0.01, ***P < 0.001.

Children with AIM were divided into three groups based on the EBV viral load. The lower detection limit of EBV DNA was 400IU/ml. The low viral load was 2.6 < LgEB-DNA < 5, medium viral load was 5 ≤ LgEB-DNA < 6, and high viral load was 6 ≤ LgEB-DNA < 8. The frequency of lymphocyte subsets differed significantly in the three groups with varied viral loads. Total T cell and CD8^+^ T cell frequencies increased in correlation with the viral load, whereas CD4^+^ T cell and CD19^+^ B cell frequencies declined. (Figure 4A). Moreover, the frequency of CD8^+^ T cells in peripheral blood was shown to be positively correlated with EBV load, whereas the frequencies of B cells and CD4^+^ T cells were found to be inversely correlated with the EBV load. (Figures 4B–D). These results indicated that the viral load of primary EBV infection was associated with CD8^+^ T cell expansion and B cell contraction.

Compared with the patients with a low viral load, an obvious tendency of increase in serum ALT, AST, LDH, and ferritin levels was observed in patients with a higher viral load, and a tendency of decrease in the platelet count was noted (Supplementary Figure S3A). Serum AST and LDH levels were positively correlated with the EBV load (Supplementary Figure S3B,C). The results suggested that EBV infection enhanced the inflammation, especially in the liver.

### Kinetic analysis of immunological events in patients with AIM after antiviral treatment

**Figure 5:**
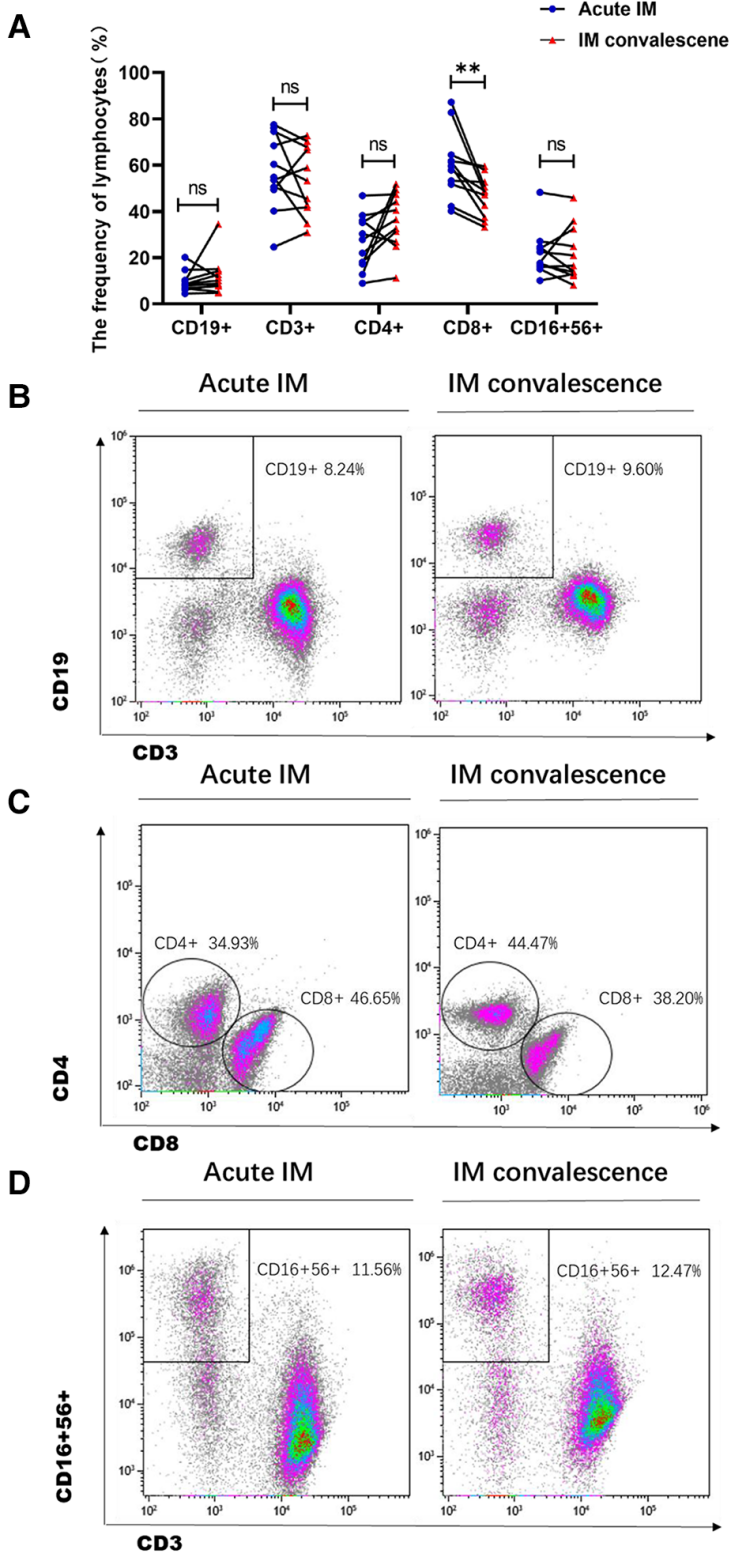
**Analysis of frequencies of the lymphocyte subsets in IM patients after anti-viral therapy.** (**A**) The frequency of lymphocyte subsets was analyzed between the acute phase and the early convalescent phase of IM disease. Representative dot plots showing the frequency of B cells (**B**), CD4^+^ and CD8^+^ T cells (**C**), and NK cells (**D**). **P < 0.01.

Among 88 AIM patients, viral load and immunological events, including EBV DNA, lymphocyte subsets, T cell phenotypes and cytokine production, etc, were kinetically analyzed in 11 patients after antiviral treatment. EBV loads declined during the first 1–2 weeks after receiving antiviral treatment (Supplementary Figure S4A). Meanwhile, after the antiviral treatment, the frequency, and the absolute number of CD8^+^ T cells significantly decreased in the peripheral blood during the early convalescent phase of EBV infection (Figure 5A,C, Supplementary Figure S4B). With a decrease in CD8^+^ T cells, the frequency of CD19^+^ B cells tended to increase (Figure S5A,B). The number of CD3^+^ T cells and NK cells decreased after the antiviral treatment (Figure 5D, Supplementary Figure S4B). Thus, the contraction of CD8^+^ T cells contributed to the restoration of CD19^+^ B cells.

**Figure 6:**
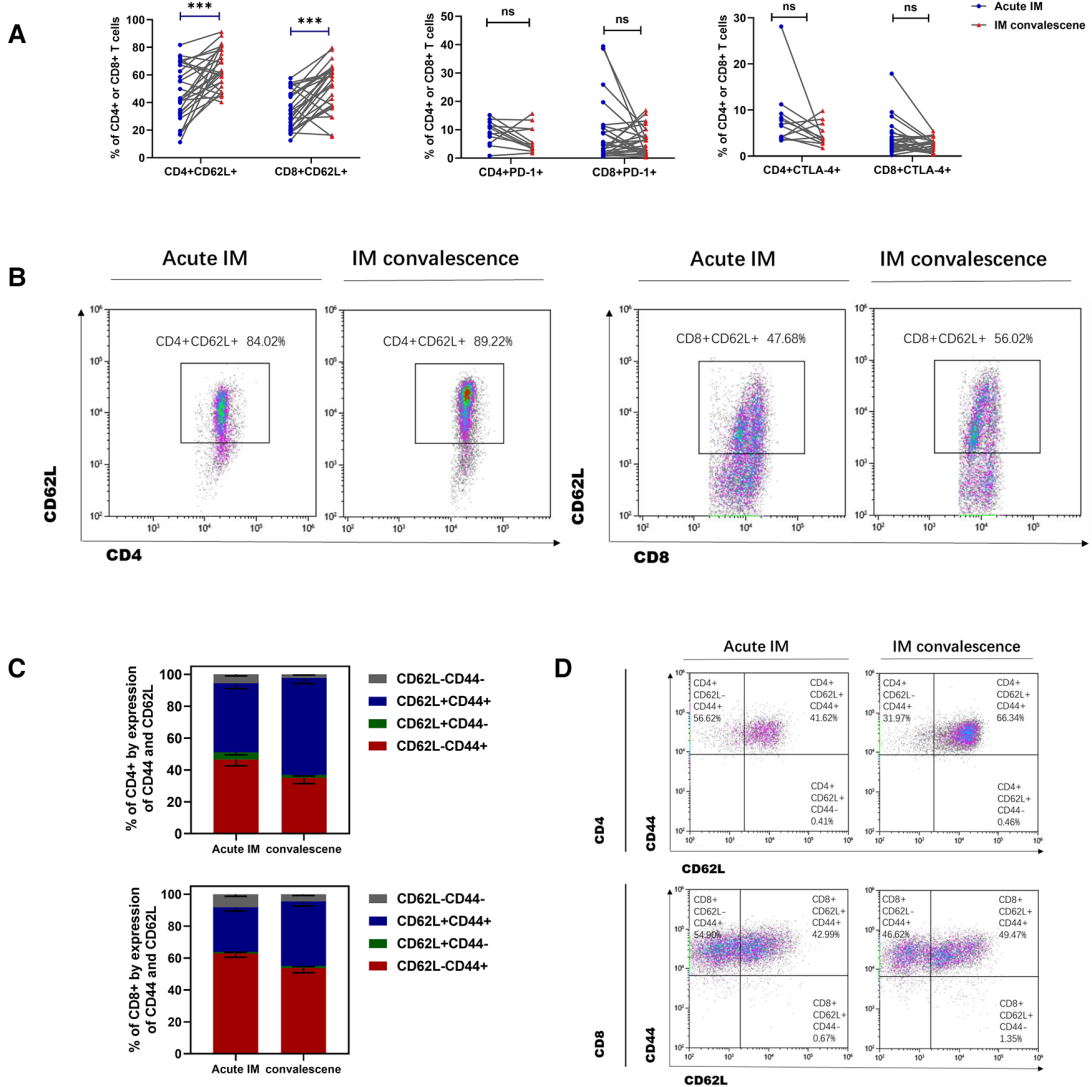
**Analysis of T cells immunophenotype in IM patients after anti-viral therapy.** (**A**) The expression of CD62L, PD-1and CTLA-4 on CD4 ^+ ^T and CD8 ^+ ^T cells was analyzed between the acute and early convalescent phases of the IM disease. (**B**) Representative dot plots showing the CD62L expression on CD4 ^+ ^T and CD8 ^+ ^T cells. (**C,D**) The frequencies of different subsets of CD8^+^ T cells and CD4^+^ T cells indicated by the CD44 and CD62L expression. Data are expressed as the means ± SD. ***P < 0.001.

**Figure 7:**
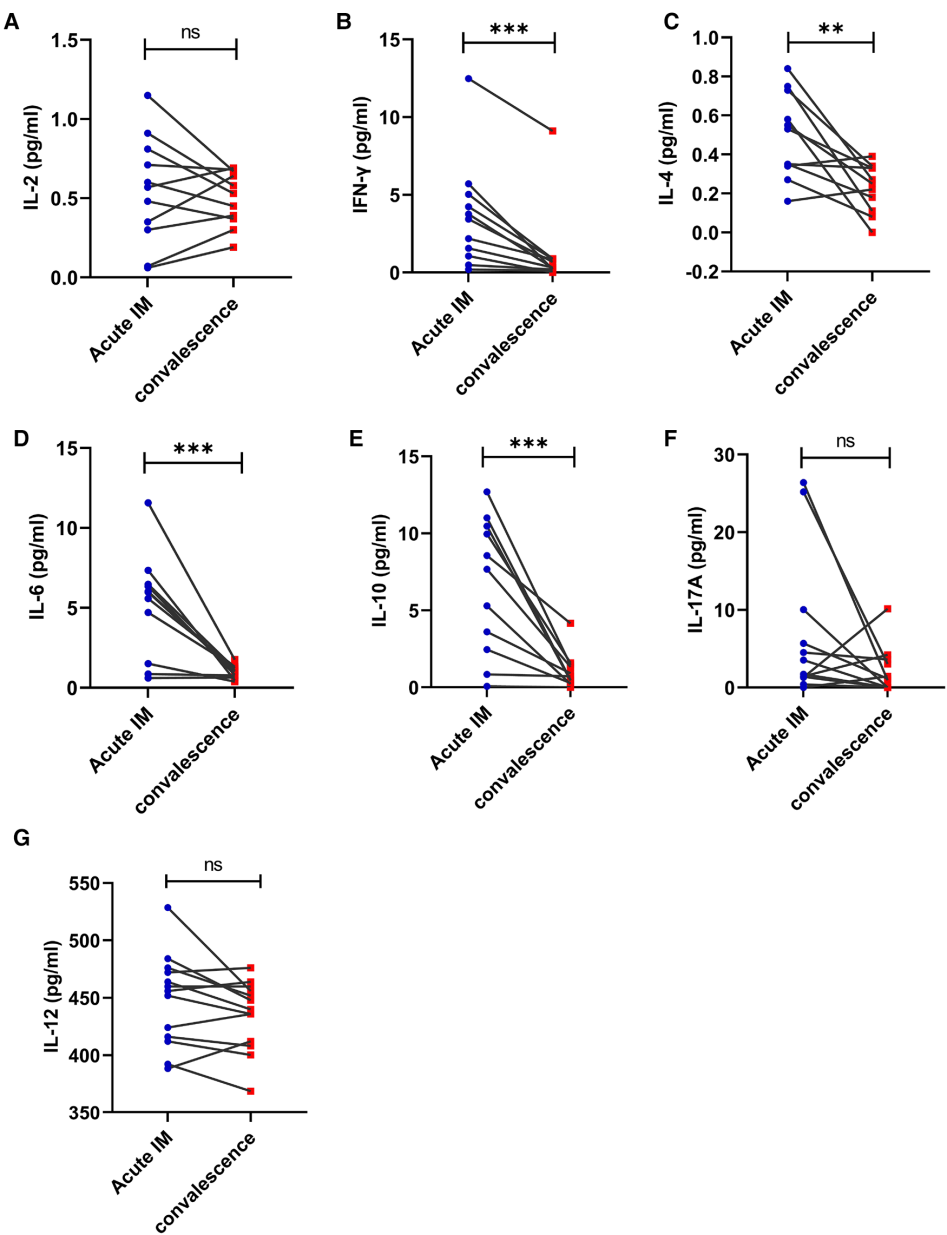
**Kinetic analysis of Th1, Th2, and Th17 related cytokines and IL-12 during different phases of EBV-infected IM disease.** The serum levels of cytokine IL-2 (**A**), IFN-*γ* (**B**), IL-4 (**C**), IL-6 (**D**), IL-10 (**E**), IL-17A (**F**), and IL-12 (**G**) were analyzed by CBA at the acute and early convalescent phases of IM. **P < 0.01, ***P < 0.001.

The immune phenotypes of T cells were also analyzed. CD62L expression on CD8^+^ and CD4^+^ T cells increased significantly in the patients with IM after 1–2 weeks of antiviral therapy (Figure 6A,B). However, no significant difference was observed in CTLA-4 and PD-1 expression on CD4^+^ and CD8^+^ T cells after antiviral therapy (Figure 6A). It was also demonstrated that the ratio of CD44 ^+ ^CD62L^−^CD8 ^+ ^T that accounted for total CD8^+^ T cells and the ratio of CD44 ^+ ^CD62l^−^CD4^+^ T that accounted for total CD4^+^ T cells reduced, whereas the ratio of CD44 ^+ ^CD62L ^+ ^CD8 ^+ ^T that accounted for total CD8^+^ T cells and the ratio of CD44 ^+ ^CD62L ^+ ^CD4^+^ T that accounted for total CD4^+^ T cells increased in the patients with IM after antiviral therapy (Figures 6C,D). The results indicated that the strongly activated T cells gradually returned to normal, which was demonstrated through upregulated CD62L expression and conversion of more effector T cells into memory T cells. The levels of plasma cytokines, the Th1 cell marker IFN-*γ*, and the Th2 cell marker IL-4, IL-6, and IL-10 were considerably lower in the patients with AIM after antiviral treatment (Figures 7A–G).

**Figure 8:**
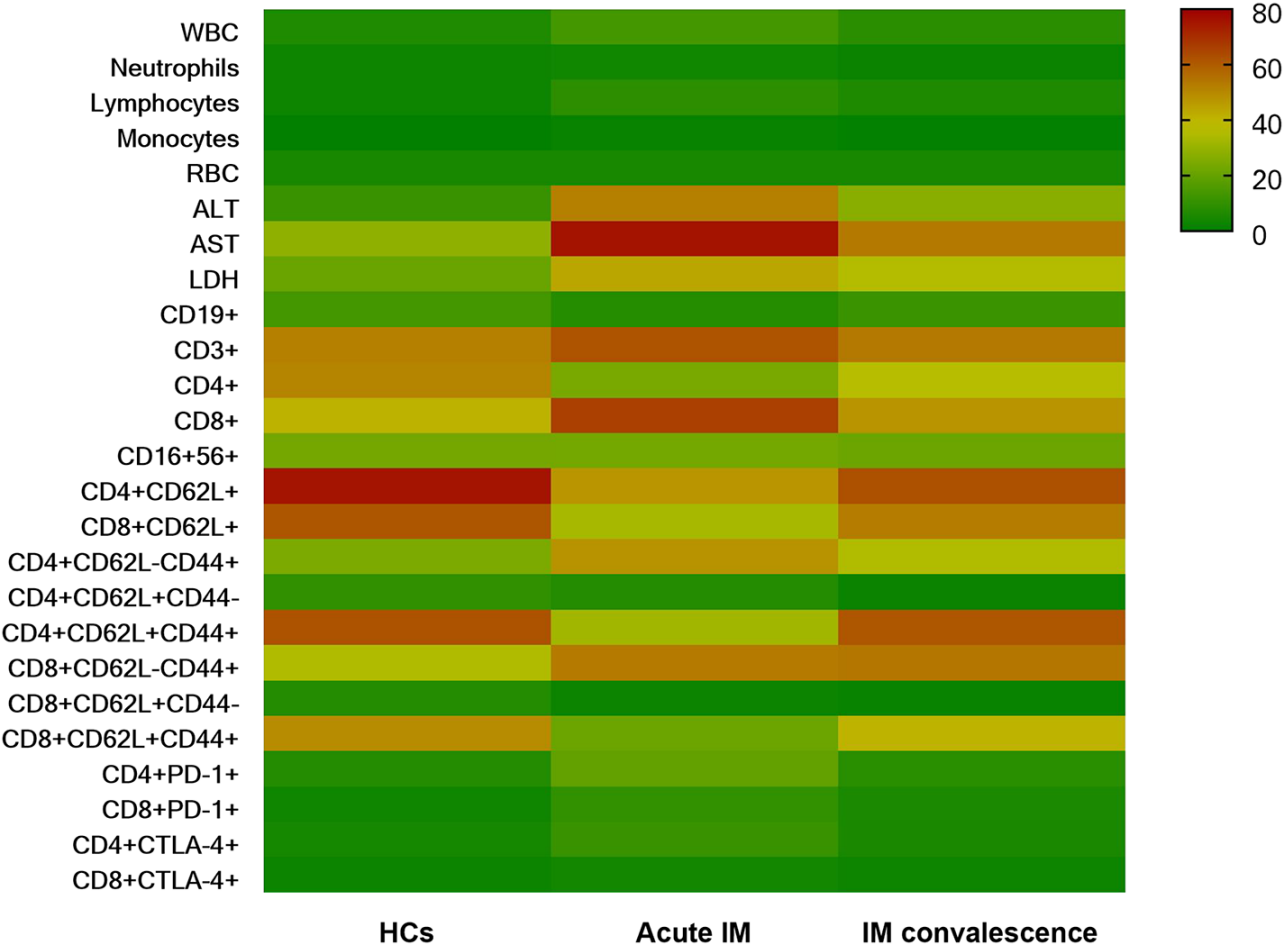
The overall characteristics of immunological events demonstrated by heatmap analysis.

A hierarchically clustered heatmap was created for sample visualization because of the various laboratory measurements, lymphocyte counts, and immunological marker expression among various individuals (Figure 8). The overall analysis of immune signatures showed that the AST level, the frequency of CD8^+^ T cells, and proportion of effector subsets in CD4^+^ and CD8^+^ T cells increased, whereas CD62L expression on CD4^+^ and CD8^+^ T cells decreased in the AIM group compared with the HC and convalescent group.

Taken together, these results suggested that the immune environment, including inflammation, lymphocyte subsets and immune response, tended to normalize during the convalescent phase in patients with IM after antiviral therapy.

## Discussion

One of the most common infections in people, Epstein-Barr virus is the primary factor in pediatric infectious mononucleosis. In our study, EBV-infected children with IM were mainly aged 1–13 years old, and the male-to-female ratio was 1.93:1, which is consistent with previous studies (13). IM is an immunopathologic condition arising due to the response of both humoral and cellular immunity to EBV. Although primary EBV infection is self-limited, immunocompromised patients may experience recurrence and significant diseases. An extremely aggressive immune response to EBV infection causes acute IM symptoms (14). During EBV infection, B cells are usually infected by the CD21 receptor, and this stimulates a robust expansion and immune response of CD8^+^ T cells to eliminate infected B cells. EBV infection control is greatly influenced by the cellular immune response, particularly CD8^+^ T cell responses. We here found that pediatric patients with IM had a significantly higher CD8^+^ T cell frequency and count, but not CD4^+^ T cell frequency or count than the HCs, which is consistent with the result of previous reports (14–16). The lymphocyte subset NK cells in patients with IM was different than previous studies. Obinna C et al. reported that NK cell frequency decreased both in the spleen and periphery (17). According to Hilary Williams et al., NK cell counts were dramatically increased both at the time of IM diagnosis and in the first month that followed (18). During EBV infection, NK cells also expand, leading to an increased NK cell count; however, the NK cell frequency does not increase, which may be attributable to the large clonal expansion of CD8^+^ T cells in children with IM.

All IM symptoms and complications are associated with T-cell activation and cytokine production (19, 20). T cells from children with AIM were highly activated by the high expression of the active markers CD43 and CD44 and decreased expression of the naïve or memory marker CD62L. CD62L is a member of the selectin family, that mainly mediates the migration of the cell in peripheral lymph nodes and is essential in the homing of lymphocytes to the lymph (21). Therefore, CD62L is mainly used for detecting effector and activated T cells, as well as for studying cell differentiation. In the present study, the ratio of effector CD8^+^ T cells increased in children with AIM, with strongly downregulated CD62L expression and increased CD44 expression. During the main response to EBV, a massive proliferation of activated T cells has been noted with of the downregulation of CD62L expression (22).

How PD-1 regulates T cell exhaustion during chronic viral infection has been extensively researched (23–25). However, PD-1 is also expressed in the early T cell activation stages and has a crucial regulatory role in the differentiation of naive to effector CD8^+^ T cells (26). During EBV infection, PD-1 blockade and CTLA-4 deficiency resulted in increased viral loads and EBV-associated severe diseases (27). Interestingly, during the acute phase of IM, PD-1 and CTLA-4 expression on CD4^+^ and CD8^+^ T cells increased significantly in children with AIM. Thus, the expression of co-inhibitory molecules on T cells, which inhibit T cell overactivation by binding with corresponding receptors, balances T cell activation and tolerance (26, 28).

Exposure to EBV leads to AIM or other EBV-related diseases. Effector T cell responses and robust and widespread T cell proliferation are both essential for EBV eradication. Initially, the direct cytolytic effector capabilities of CD8^+^ T cells were believed to contribute to virus clearance. Noncytolytic inhibition of EBV replication also contributes to virus control. Upon activation, CD8^+^ T cells produce cytokines, such as IFN-*γ*, IL-2 and TNF-α (29). Granzymes (Gzm) are a family of serine proteases detected in the secretory granula of cytotoxic T cells or NK cells upon their activation. In this study, granzyme B production by CD8^+^ T cells increased, but that by NK cells decreased. By contrast, IFN-*γ* secretion by CD8^+^ T cells decreased, whereas that by NK cells increased. According to the results, noncytolytic and cytolytic effector functions of CD8^+^ T cells and NK cells are regulated in an oscillatory manner, which may be due to different EBV antigen recognition and the upregulation of the co-inhibitory molecules PD-1 and CTLA-4. During EBV infection, such oscillations may coordinate the immunological events, thereby minimizing tissue injury and maximizing virus control (12).

AIM mostly occurs in children, and the disease severity varies. Therefore, searching for indicators of evaluation is of great significance. The EBV load and host immune response are understood to be essential for disease severity. A previous study indicated that EBV loads increased with IM severity (30). The level of inflammatory markers such as AST and LDH, and the frequency of CD8^+^ T cells were positively correlated with the viral load, whereas the frequencies of B cells and CD4^+^ T cells were negatively correlated with viral the load. Therefore, it is speculated that AST, LDH level and the frequencies of B cells, CD8^+^ T cells and CD4 ^+ ^T cells were crucial in evaluating the severity of EBV-related IM in patients.

Although antiviral treatment has been applied for IM, more firm evidence is required to support its use (31). To monitor the efficacy of antiviral treatment, kinetic changes in lymphocyte subsets, T cell phenotypes, and general laboratory indicators were analyzed in patients with AIM from the onset of disease to the convalescent phase. After antiviral treatment, the frequency of CD8^+^ T cells decreased and that of CD19^+^ B cells tended to restore; highly activated CD8^+^ and CD4^+^ T cells gradually returned to normal. In these patients, the general laboratory indicators also became normal, including WBC, lymphocytes, and AST and LDH levels etc. Thus, the efficacy of antiviral therapy for IM was confirmed to a certain extent.

This study illustrated the immunological characteristics of EBV infection in children with IM. However, there were also some limitations. For NK cells analysis, we use the same fluorescent label for CD16 and CD56 antibody. Therefore, NK cells cannot be divided into CD56^bright^ and CD56^dim^ population in which CD56^bright^ population produces large amounts of IFN*γ* and CD56^dim^ is more cytotoxic. In our study, total NK cells showed increased IFN*γ* production, but decreased granzyme B secretion which may be due to the increased CD56^bright^ NK cells and decreased CD56^dim^ population. And it is better to expand the sample size for dynamic analysis of antiviral efficacy.

In conclusion, this study provided the features of immunological events in EBV-infected children with IM. Robust expansion of CD8^+^ T cells contributed to lymphocytosis. T cells were activated by the high expression of active markers CD43 and CD44 and downregulated CD62L expression, which was also regulated by the upregulated expression of inhibitory molecules PD-1 and CTLA-4 to prevent T cell overactivation. Noncytolytic and cytolytic effector functions of CD8^+^ T cells and NK cells are regulated in an oscillatory manner, which minimizes tissue injury and maximizes the virus inhibition. The assessment of immunological events in patients with IM not only furthers our understanding of EBV pathogenesis but also adds value to disease severity evaluation and guides therapy for the disease.

## Data Availability

The raw data supporting the conclusions of this article will be made available by the authors, without undue reservation.
