# Supplementary material for: Characteristics of immunological events in Epstein-Barr virus infection in children with infectious mononucleosis
Source: Front Pediatr. 2023 Feb 9;11:1060053. doi: 10.3389/fped.2023.1060053 (PMC9949895; doi:10.3389/fped.2023.1060053)
Supplement: Supplementary file 1 [file Datasheet1.pdf]

Supplementary Material

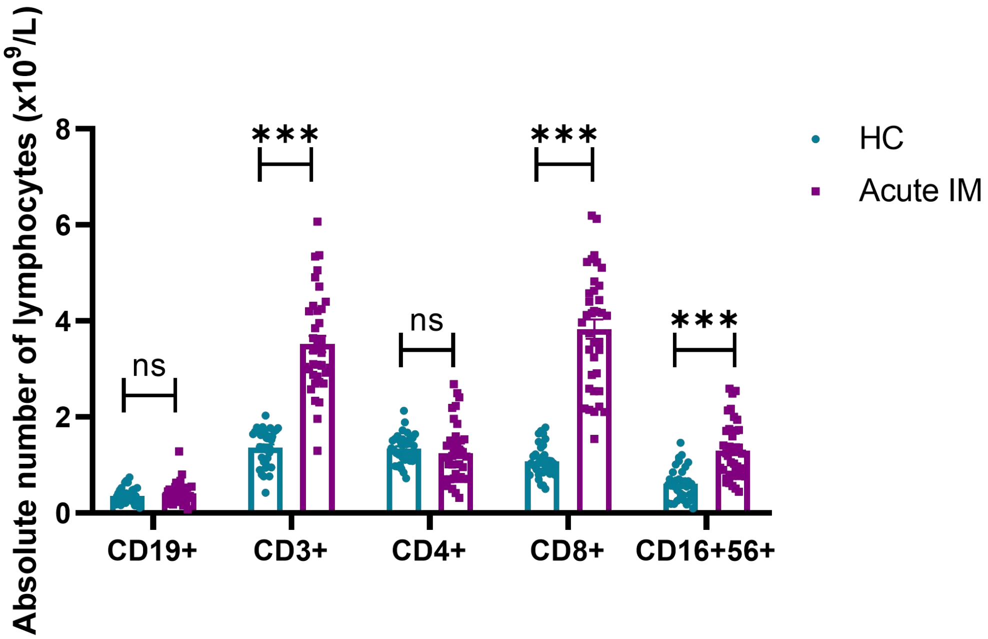

Supplementary Figure 1. The absolute number of $CD19^{+}$B cells, $CD3^{+}$ T cells, $CD4^{+}$ T cells, $CD8^{+}$ T cells and NK cells in pediatric patients with infectious mononucleosis (IM) and healthy controls were analyzed. Data are expressed as the means±SD. ***$P < 0.001$.

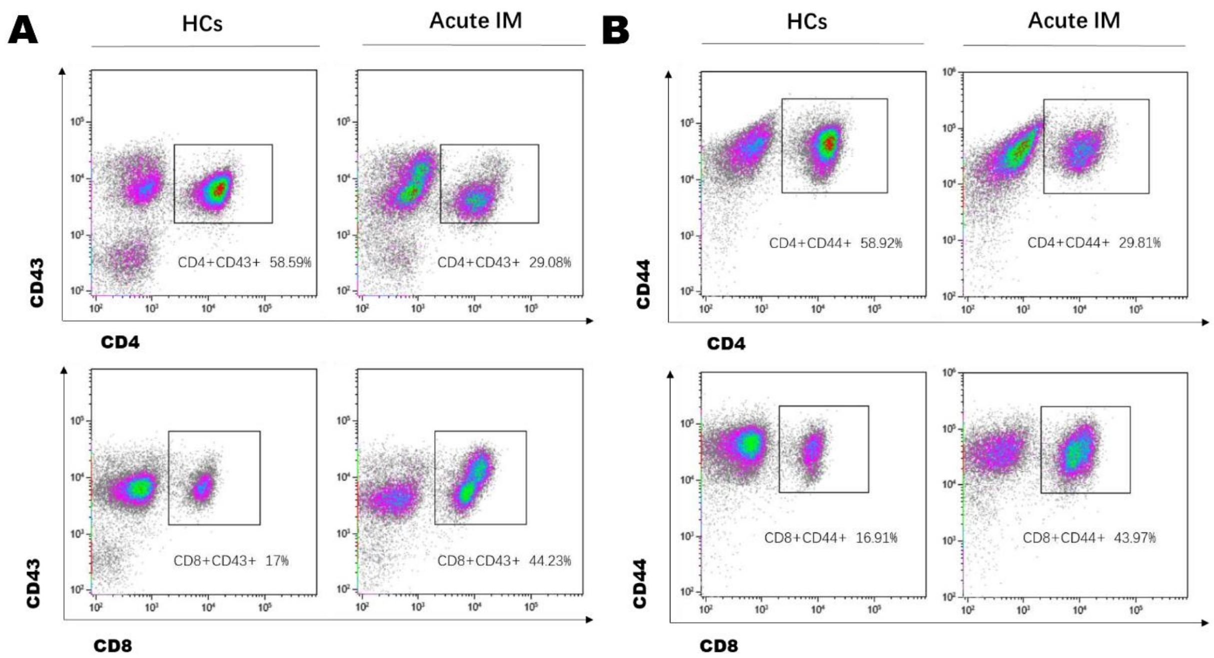

Supplementary Figure 2. Representative dot plots depicting the active markers CD43 (**A**) and CD44 (**B**) expression on $CD4^{+}$T and $CD8^{+}$T cells.

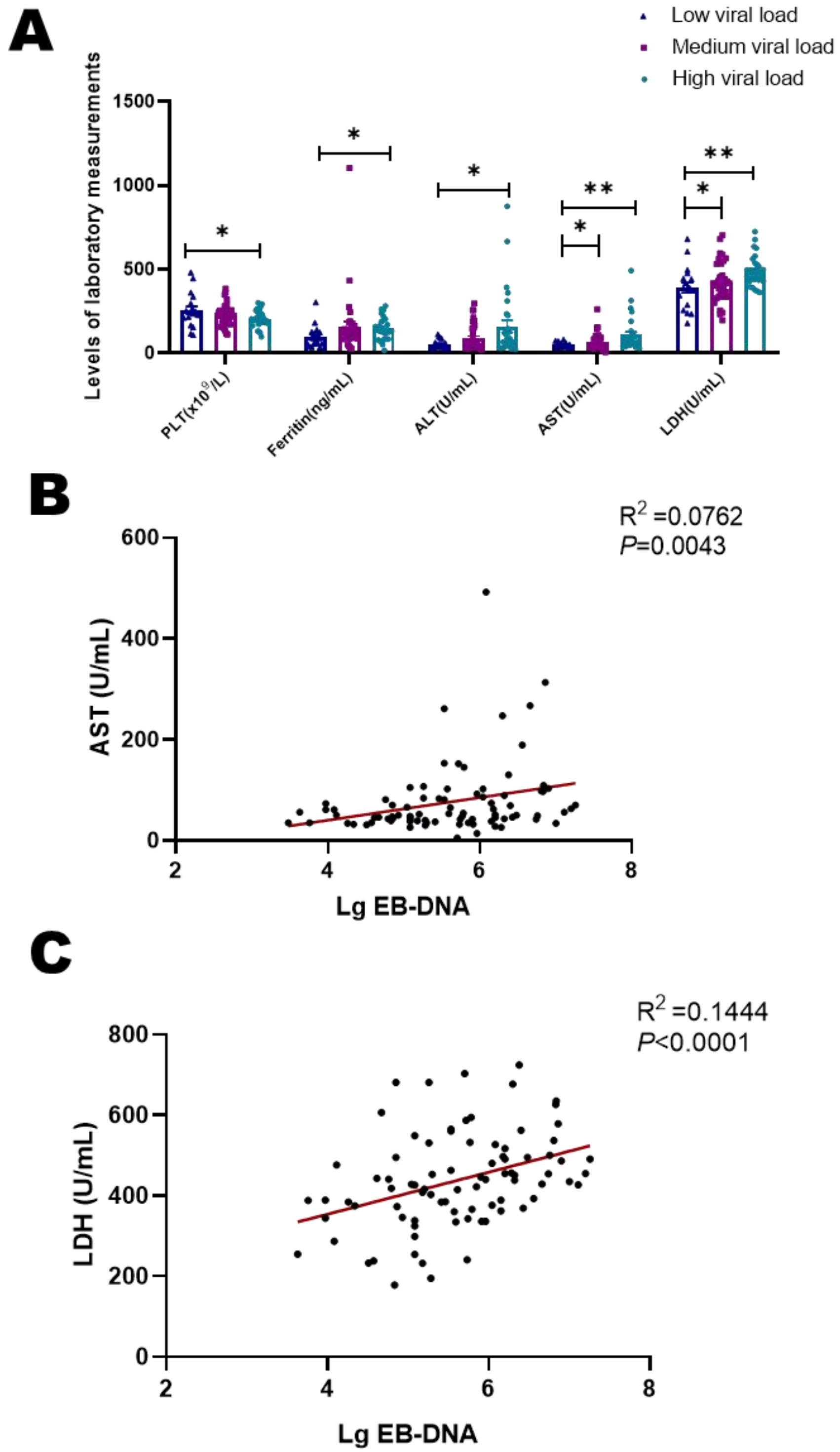

Supplementary Figure 3. Analysis of the correlation between EB viral load and general laboratory measurements. (**A**) The serum level of PLT, ferritin, ALT, AST and LDH in different viral load groups. Correlation between the EB viral load and AST (**B**) and LDH levels (**C**) were displayed. Data are expressed as the means±SD. * $P < 0.05$, ** $P < 0.01$.

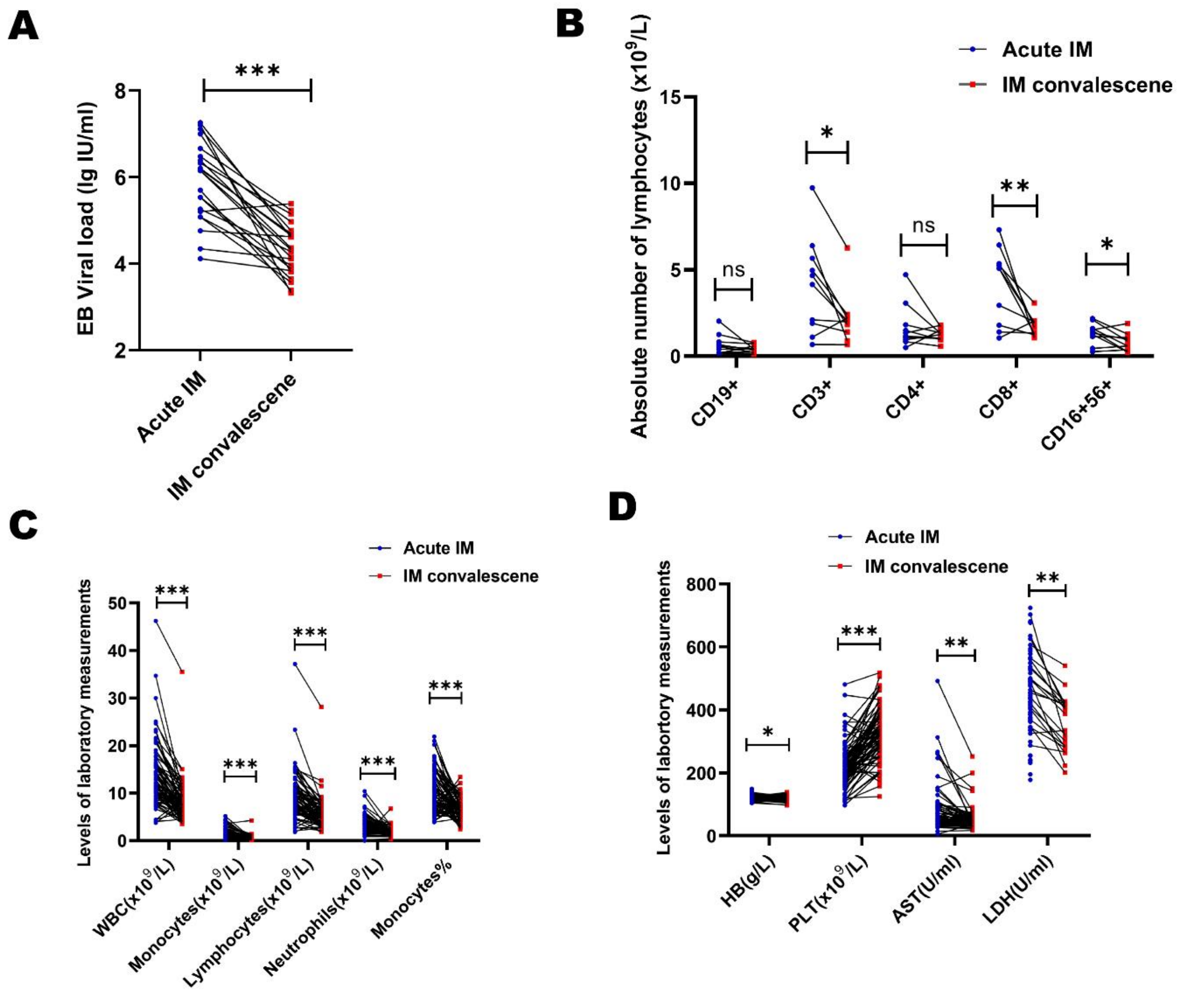

Supplementary Figure 4. The restoration of EBV viremia (**A**), the absolute number of different lymphocytes (**B**), blood routine test including the enumeration of WBC, monocytes, lymphocytes, neutrophils, and monocyte frequency (**C**), as well as general laboratory measurements including HB, PLT, AST, and LDH (**D**). Data are expressed as the means±SD. *$P < 0.05$, **$P < 0.01$, ***$P < 0.001$.

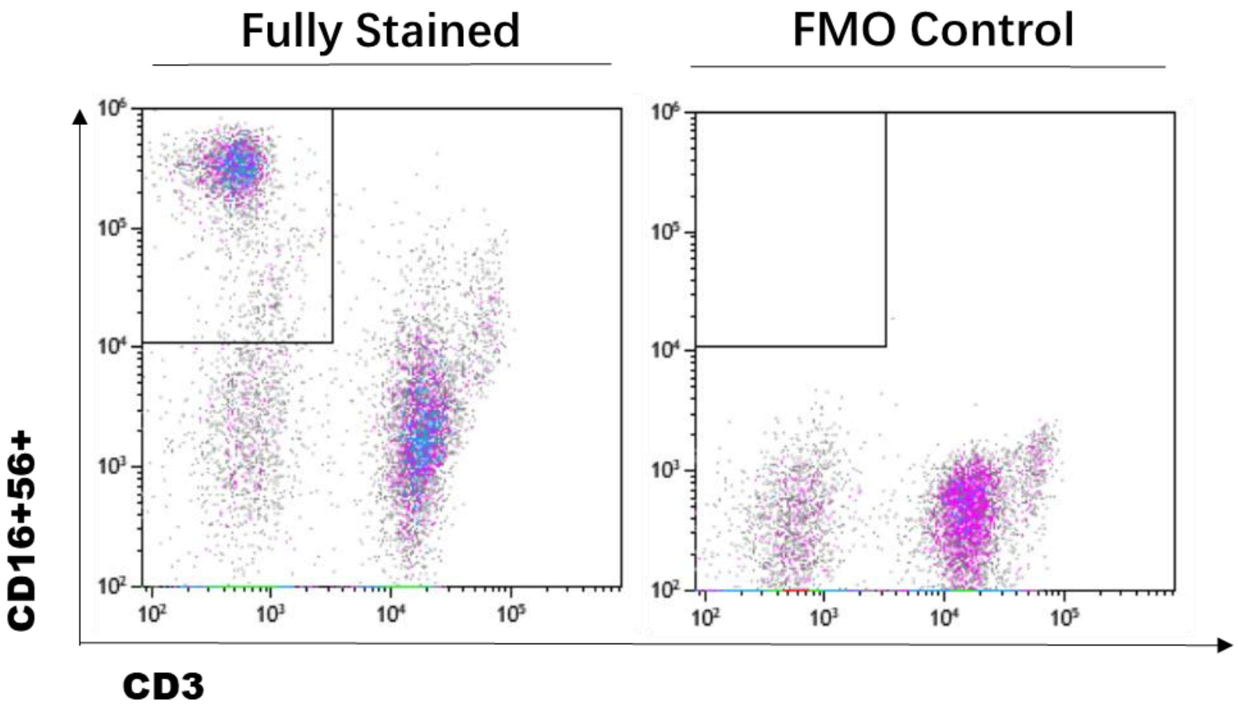

Supplementary Figure 5. Flourescence-minus-one (FMO) control for gating CD16^{+}56^{+}.
